# Supplementary material for: Are carbon emissions trading and green financial instruments synergistic? -Comprehensive quantitative research based on content analysis
Source: PLoS One. 2024 Mar 7;19(3):e0298601. doi: 10.1371/journal.pone.0298601 (PMC10919601; doi:10.1371/journal.pone.0298601)
Supplement: S1 Data — (ZIP) [file pone.0298601.s001.zip › Data/地级市Z_F工作报告.zip!/╔┬╬≈/░▓┐╡╩╨/└┤╫╘╣1⁄2╓┌║┼í╛┬φ┐╦╩2╛▌═°í┐_2007.docx]

各位代表：

　　现在，我代表市人民政府，向大会作工作报告，请予审议。并请市政协委员和其他列席人员提出意见。

　　一、2006年工作的回顾

　　刚刚过去的一年，我们在中共安康市委的正确领导下，坚持以科学发展观统揽全局，紧紧依靠全市人民的智慧和力量，艰苦奋斗，开拓进取，完成了市二届人大一次会议确定的目标任务，实现了“十一五”经济社会发展的良好开局，绿色产业与和谐社会建设迈出了坚实的步伐。

　　经济保持较快增长。在酝酿绿色产业的突破中，全市经济呈现良好发展态势。生产总值达到157.2亿元，增长10.3％；财政总收入12.8亿元、地方财政收入4.6亿元，分别增长20.2%和21％；社会消费品零售总额完成53亿元，增长14％；农民人均纯收入1953元，增长8.6%；城镇居民人均可支配收入6860元，增长7.4%。社会主义新农村建设推进了“三农”工作的落实，广大农村的产业建设、基础设施、村容风貌、社会事业正在发生着深刻变化。在严重干旱的情况下，粮食总产95.7万吨，油料产量7.7万吨。生猪饲养量362万头、出栏186万头，分别增长14.6％和19％；养蚕发种50.3万张，蚕茧产量1.8万吨，分别增长6.5％和9.7％。劳务输出46.7万人，劳务收入20.8亿元。成林油脂、民荣食品、秦东魔芋、宝业与巴山丝业、阳晨生猪繁育中心等企业的发展壮大，标志着农业产业化中的龙头建设取得实质性突破。以通村水泥路为重点的农村基础设施建设成效显著。铺筑通村水泥路1715公里，超额完成省上下达的1300公里计划，惠及541个村80万人。黄石滩水库下闸蓄水。新修农田4万亩。造林绿化38万亩，工程造林13.8万亩。饮水解困76000人。新修沼气池1.5万口。102个新农村建设试点村起到了示范作用，453个扶贫重点村建设扎实推进，131个特困村脱贫攻坚得到重视，农业综合开发取得实效。国企改革和骨干项目建设有力推动了工业发展。昊华和江华钡盐建成投产，使我市成为全国重要的钡盐化工基地。喜河电站提前一年发电。以绿色资源为依托的工业开发取得长足进步，民营资本投资工业的力度加大，中小企业发展的后劲和活力明显增强。全年实现工业总产值83.6亿元，其中规模以上工业产值54.3亿元，分别增长16％和22.4%。生态旅游继续保持良好态势，全年接待游客160万人次，综合收入5.6亿元。市场繁荣活跃，物价基本稳定，金融存贷两旺，交通通讯持续增长。税务、电力、通信、金融、保险、铁路、民航、石油、烟草等行业为地方经济发展做出了新的贡献。

　　重点项目扎实推进。“项目建设年”卓有成效，全年共完成全社会固定资产投资72亿元，增长16％。铁路、高速公路等中省重大项目完成投资48.8亿元。汉江水电梯级开发全面启动，小水电新增装机2.4万KW，安喜汉330KV输电工程、330KV金州变电站建成运营。20个地方重点工业项目完成投资2.51亿元。教育、卫生、文化等社会事业项目完成投资2.98亿元，超过历史任何一年。中心城市建设水平提升，当年完成投资3.02亿元。修编完成了江南城区改造和江北新区建设规划，以汉江三桥、安康大道、滨江大道为重点的江北新区基础设施建设全面开工；以汉江公园建设、大桥路强电入地、育才西路和南环路西段拓宽改造为重点的江南综合改造工程基本竣工；以突出山水、彰显绿色为特征的县城和重点集镇建设呈现新的亮点。进一步加强南水北调水源地保护，小流域治理、荒山造林、退耕还林、天然林保护等生态建设项目扎实推进，大瀛湖生态环境治理工程首战告捷，江南污水处理厂和江北垃圾处理厂建成投运。汉江水质保持Ⅱ类水平，受到国务院领导和省委、省政府的充分肯定。

　　改革开放取得实效。国企改革实现了预期攻坚目标，40户国有企业成功改制，尤其是丝一厂、丝二厂、安运司等重点国有企业的改制重组，标志着国有经济从竞争性领域有序退出的任务基本完成。非公有制经济蓬勃发展，全市新增工商企业902户，其中工业企业146户。以党政机关和事业单位资产的统一管理、统一配置、统一经营为主要内容的非经营性国有资产管理改革，在探索中不断深化，产生了良好的综合效益，被财政部总结为“安康模式”。本着互惠共赢、风险共担的原则，与国家开发银行搭建了政府、银行、业主投融资的平台，走出了一条破解市政建设和中小企业资金难题的路子。开拓了财信担保、典当业务，增强了政府为中小企业和民营经济服务的能力。以推进乡镇机构、农村义务教育、县乡财政管理体制为主要内容的农村综合改革全面展开。粮食流通体制改革基本完成。收入分配制度改革平稳推进，《公务员法》的实施工作进展顺利。对外开放迈出新步伐，招商引资取得新成果。全年共签订引资合同项目110个，总投资39.86亿元，到位资金24.58亿元。香港建溢集团整体开发旬河水能资源，昊华化工集团在平利建设钡盐基地，广东核电集团投资白河电站，湘火炬集团开发生物能源，中国华电集团和中电投资集团进入火电项目前期工作，标志着我市的招商引资转向大企业、大项目，对外开放的层次进一步提升。

　　社会事业协调发展。坚持统筹发展的思想，更加重视社会事业。全市财政投入社会事业建设10.2亿元，比上年增长15％。科技对经济增长的贡献率显著提升，以蚕桑、畜牧、茶叶、粮油为重点的农业适用技术大面积推广，以设施农业和良种繁育为重点的月河农业科技示范园建设，以黄姜皂素清洁生产、股兰泼尼松研发、魔芋仿生食品开发、钡盐加工为重点的产品和工艺创新，推动了增长方式转变和产业升级。教育优先发展的地位进一步巩固，基础教育得到高度重视，“两基”成效充分显现，新增中小学危房改造和“两免一补”资金16398万元。高考取得好成绩。市区教育资源整合扎实起步。发展职业教育的办学思想进一步明确，专业设置进一步适应市场需求，新型劳动力培训力度进一步加大，各类职业技术学校当年毕业7702人，就业率达到95％，劳务培训13000人。安康学院的建立，安康职业技术学院的组建，刷新了安康高等教育的历史，在校本专科学生达到7321人。公共卫生事业的加快发展，改变了长期以来农村医疗服务薄弱的状况，面对广大农民的医疗服务机构全部纳入财政供给，新改建基层卫生院65所。疾病防控体系建设和重大传染病、地方病防治工作得到加强，儿童计划免疫率达90％以上。以“三清三查”为重点的计划生育工作扎实有效，全市继续保持低生育水平。以“八荣八耻”为重点的社会主义荣辱观教育广泛开展，群众性精神文明创建活动不断深入，城乡各类文体活动蓬勃开展，文艺创作日趋活跃，紫阳民歌、汉调二簧被国务院列为非物质文化遗产目录，我市在全省十三届运动会上取得良好成绩。外事、民族、宗教、气象、地震、档案、方志、老龄、残疾人和妇女儿童等各项事业取得新进步。慈善协会、红十字会扶弱济困的作用更为突出。

　　高度重视民生问题。更加关注民生，公共财政更多地用于改善困难群体的生产生活。全年新增就业和再就业11000人。财政安排5.45亿元投入到城乡社会保障，城市低保42600人，农村低保71500人。投资1000万元建成了87所敬老院、249个五保户集中安置点，集中供养五保户3000人。职工基本养老、失业、医疗保险体系不断完善。农村义务教育“两免一补”政策得到全面落实，免除杂费、教科书费和兑现寄宿生生活补助费共10830万元。贫困家庭大学生全部得到资助顺利入学。旬阳、石泉、平利、白河四县842296人参加了新型农村合作医疗，参合率达90％，共有102454人报销医疗费2889万元。投入1300万元防氟改灶6.5万户。制定并启动了安康城区廉租房三年建设规划，首批住房困难户可于春节前领到新房钥匙。背街小巷改造转向居民片区综合配套设施建设，公厕、照明、给排水、保洁逐步到位。当年新建便民桥336座，三年累计建成1436座，超额完成了1000座修建任务。

　　社会保持稳定和谐。坚持人民利益高于一切、安全稳定压倒一切，努力增强政府应对突发事件和防范事故灾害的能力。编制完成了公共突发事件应急预案。扎实进行道路交通、煤矿和非煤矿山专项整治，有效遏制了重特大事故发生。认真开展“平安安康”创建活动，健全群防群治网络，依法打击各类犯罪活动，一批重大案件的快速侦破，震慑了犯罪，安定了民心，维护了社会稳定。驻安解放军、国家安全、公安、武警为保卫改革开放、维护人民生活安宁，做出了积极贡献。加大信访积案排查力度，认真落实领导包案、信访接待日制度，办结了一批信访积案要案。廉政建设进一步加强，扎实治理商业贿赂，严肃查处损害群众利益的不正之风，着力整治投资环境，有力推进了政风行风建设。认真执行人大及其常委会的各项决议决定，自觉接受人大依法监督、政协民主监督、新闻舆论监督，大力支持检察、监察、审计的法纪政纪监督。倡导勤政务实之风，切实改进领导作风，重视基层基础工作，各级政府的服务意识和行政效能有了新的提高。

　　回顾过去的一年，我们每一个微小的进步、每一项事业的发展，无不归功于科学发展观的正确指引。实践使我们更加深刻的体会到，在实施突破发展中，必须始终重视安康的长远利益和未来发展，实现绿色资源的有效积累和合理利用；在面临诸多挑战的压力下，必须始终着眼于夯实基础、统筹发展，绝不急功近利、心浮气躁；在财力依然困难的情况下，必须更多地关注民生，重视社会事业发展，维护公平正义。

　　各位代表！2006年工作取得的成绩，是中共安康市委正确领导，市人大、市政协监督支持，全市人民和各条战线团结奋斗的结果。在此，我代表市人民政府,向全市广大工人、农民、知识分子和各级干部，向关心和支持政府工作的人大代表、政协委员、各民主党派、人民团体、离退休老同志和社会各界人士，向驻安人民解放军、武警官兵和政法干警，向所有融入安康发展的外来投资商和国内外关心家乡建设的安康儿女，致以崇高的敬意和衷心的感谢！

　　在前进的道路上我们还面临着一些突出的困难和问题。主要是工业基础薄弱，增长方式粗放；基础设施的瓶颈制约尚未根本缓解，财政供需矛盾依然突出；就业压力日益增大，扶贫攻坚任务还十分艰巨；人民群众对官僚主义、形式主义、作风飘浮、奢侈浪费的问题反映十分强烈；投资环境还需下力整治，“吃拿卡要”在一些地方和单位还很严重，损害群众利益的行为时有发生，政府工作人员的勤政廉政建设还要进一步加强。这些都需要我们高度重视，切实加以解决。

　　二、2007年工作的目标任务与总体要求

　　2007年，政府工作的总体要求是：以科学发展观为指导，认真落实市第二次党代会精神，积极推进社会主义新农村建设，着力培育工业支柱产业，切实加强基础设施和生态环境建设，不断提升城镇建设管理水平，推进科技进步，深化改革开放，转变经济增长方式，统筹发展社会事业，努力改善人民生活，奋力实现绿色产业新突破，迈出和谐安康建设新步伐。

　　经济社会发展的预期目标是：生产总值达到175亿元，增长10%；全社会固定资产投资85亿元，增长15%；财政总收入14.8亿元，增长15%，其中地方财政收入5.6亿元，增长21％；城镇居民可支配收入净增500元，农民人均纯收入净增150元；城镇登记失业率不超过4.5％；人口自然增长率控制在3.5‰以内；单位生产总值能源消耗降低4.5%；汉江安康段水质保持Ⅱ类水平。

　　实现今年发展目标，必须准确把握以下指导原则：

　　（一）坚持项目带动战略。项目建设决定突破发展。无论是争取投资、还是吸引外资，无论是当年增长、还是积蓄后劲，都必须建立在项目支撑的基础上。牢固确立项目是全局的意识，坚持用项目建设评价发展，用项目质量和效益衡量领导经济工作的水平。

　　（二）坚持基础建设先行。实施突破发展首先在于基础建设突破。要立足大局，抓住机遇，以机场改造、铁路复线、高速公路建设为契机，突破对外开放的瓶颈，倾力抓好城乡交通、市政设施、农田水利、防洪保安工程，夯实加快发展的基础。

　　（三）坚持城乡统筹发展。市情特征要求我们在绿色产业的突破中，必须以绿色资源为依托，以产业基地为基础，以药水游为重点，以龙头企业为纽带，更加自觉地把工业与农业、城市与农村、富民与强市统筹起来，用工业化牵引产业化，推进城镇化，实现城乡协调发展。

　　（四）坚持绿色增长方式。绿色是我们的特色，是生存和发展之本。在绿色资源的开发建设中，绝不能以粗放经营、破坏生态、牺牲绿色为代价，坚持走新型工业化道路，高度重视节能降耗减排，实现集约化经营、环保式生产、可持续发展。

　　（五）坚持以人为本理念。发展依靠人民，发展为了人民，发展成果人民共享，是构建和谐安康的核心。要更加关注民生，更多的提供公共产品，更好的关顾困难群体，实现好、维护好、发展好人民群众的根本利益，促进人的全面发展。

　　三、奋力实施绿色产业突破发展

　　我市的绿色产业主要是指秦巴汉水独特的自然环境下形成的生物、水能、矿产、旅游四大资源的积累、开发与保护。资源多样性、广分布和生产力水平的多层次，决定了绿色产业的发展，必须立足富民，多元发展，强化基础，重点突破。

　　（一）夯实产业基础，扎实推进新农村建设。坚持以新农村建设总揽农村工作全局，把“三农”工作放在重中之重的地位。以农民增收为重点，夯实绿色产业基础。巩固粮食生产能力，粮食播种面积稳定在490万亩，总产保持90万吨的水平。把绿色产业基地建设切实转向“龙头牵引、强村大户、依靠科技、集约经营”的轨道。扩大食用油料生产，建设稳固的油品原料基地；主攻20个强乡镇、400个专业村、5000个生猪养殖大户，建设月河川道绿色瘦肉型生猪基地，全市出栏生猪210万头；提升40个乡镇、50000个养蚕专业户的规模经营水平，建设“四个翻番”的稳固基地，今年新增发种10万张，蚕茧总产达到2万吨；突出基地扩张、品牌整合、精深加工、品质提升，做大做强富硒名茶产业，总产达到5000吨；稳步扩大GAP药源基地，巩固提高传统地道中药材的质量和收益，扎实抓好适销对路的短线中药材生产，大力发展订单农业，拓展销售渠道；加快发展市场旺销的牛羊、家禽、水产、魔芋、烤烟、蔬菜、果品等特色产品。通过绿色产业基地建设，建立农民稳定增收的长效机制。以基础建设为先导，改善农村生产生活条件。实行最严格的耕地保护制度，坚持不懈地抓好以“小工程、大群体”为重点的水利与农田建设；继续抓好通村水泥路建设，全年新修1500公里，加大财政对边远贫困村修路的补助；解决10万人饮水困难；积极推广沼气等清洁能源，新修沼气池15000口；加快电力“户户通”、广播电视“村村通”工程建设。加大扶贫攻坚力度，切实改善人均纯收入在625元以下的131个村、52000特困农民的生存状况，启动170个扶贫重点村建设，继续抓好农业综合开发。稳定劳务输出规模，提高劳务经济质量，整合培训资源，强化技能培训，加强劳动维权，逐步实现由体力型向技能型转变。以现代农业理念为指导，积极推进现代农业建设。加大设施农业、先进技术推广、农民技能培训力度，依靠适用技术、先进设施和新型农民，奠定高产、优质、高效、生态、安全的绿色产业基础。农民是新农村建设的主人，要立足艰苦创业，主要依靠自己的努力建设美好家园，真正把建设的重心放在基础设施和主导产业上。各级政府要毫不动摇的加大对新农村建设的支持，尊重农民的意愿、尊重农民的劳动创造，既尽力而为，又量力而行，把新农村建设扎实推向前进。

　　（二）做强龙头企业，强力推动工业快速发展。绿色产业基地建设和产业龙头的崛起，为推进工业化进程创造了条件。要把工业发展放在绿色产业突破发展的中心地位，从壮大绿色产业的龙头企业入手，强力支持民荣食品、秦东魔芋、成林油脂、昊华和江华钡盐等企业尽快达产达效，竭力推动安康正大、北大制药、宝业和巴山丝业、旬阳烟厂、泸康酒业、通用机械等企业的产品创新和市场开拓，着力抓好昊华钡盐二期、石泉钒钛磁铁和旬阳鑫源选矿等重点企业扩能技改项目。制药业要增强开拓意识，下气力创新自主品牌，增强市场竞争力。茧丝业在完善体制、健全机制的基础上，重振安康茧丝品牌声誉，向深度开发迈进。要把绿色食品打造成地方特色工业的名片，在仿生魔芋食品等一两个品种上率先突破。全面推进汉江梯级水电建设，喜河电站全面竣工，蜀河电站加快建设，白河、旬阳电站正式进点，全力争取30万吨生物能源项目的审批立项，积极推进6×60万KW大型火电项目的前期工作，使安康成为新型的水火生物能源基地。在保护环境、节约资源的前提下，积极发展矿产加工业，进一步加大矿产资源的探查力度，在金、铜、磁铁等稀缺和大型金属矿种上尽快取得进展，对已基本探明的宁陕黑金彩钻花岗岩、岚皋菊花大理石、紫阳瓦板岩优质石材和重晶石、毒重石、铅锌、钼等资源，通过引进大企业，实行高起点、高环保、深层次开发。整合矿业资源、整顿矿山秩序，提升现有企业的加工能力和工艺水平。充分行使政府对矿产资源的所有权，坚决遏制无序开采，严格禁止原矿出境。高度重视中小企业的培育和发展，切实保护具有鲜明地方特色的传统产品。理顺工业园区的管理体制，发挥在工业发展中的集聚、孵化、示范作用。加快工业发展，必须从实际出发，把我们的愿望与国家的要求紧密结合起来，走出一条既符合国家产业政策，又给人民带来实惠的又好又快的工业发展路子。

　　（三）培育旅游精品，努力提升服务业水平。突出秦巴汉水、休闲度假、饮水思源主题，依托安康山水园林城市，培育以瀛湖为重点，汉江梯级库区水面相连的汉水风光游；以秦岭南麓原生态为重点，融入秦岭中国国家植物园的森林体验游；以南宫山、千家坪和鸡心岭为重点，联结渝鄂周边景区的巴山风情游。当前要重点抓好景区规划、景点开发、基础设施、服务体系建设，以最优惠的条件引进大企业、大集团高投入开发、高起点建设、高水平管理。创新“龙舟节”办节方式和内容，增强特色，提升品位，为扩大开放、经贸交流搭建平台。加强与周边地区的合作和宣传促销，扩大生态旅游的知名度，力争全年接待游客180万人次。坚持市场运作、企业经营、政府监管，深化旅游管理体制和经营机制改革。大力发展和提升批发零售、宾馆餐饮、交通运输、文化娱乐、金融保险、信用担保、中介咨询等服务业，积极培育经贸会展、物流配送、电子商务、现代信息等新型业态，不断提升服务业水平。

　　（四）突出重点项目，全力加快基础设施建设。扎实推进基础设施、绿色产业、生态环境、社会事业四大类25个重点项目建设，认真做好20个重大产业类项目的前期工作。全力组织实施省政府扶持的陕南突破发展项目。强化协调服务和环境保障，确保安康至武汉、安康至重庆、安康至西安铁路复线，西汉高速宁陕段、小康高速、安康至陕川界高速、十天高速安康段等国家重大交通项目顺利实施。开工建设安岚二级公路，修建通乡油路246公里、通达工程200公里，新增12个通油路乡镇、234个通水泥路村。扎实做好安康机场改造前期工作。加快建设330KV平利输变电工程，110KV西郊、五里和筒车湾输变电工程。进一步加大对教育、卫生、文化、体育等社会公共事业的投资，加快文化艺术中心、影剧院、新闻大厦、基层卫生院等项目建设。

　　（五）强化规划地位，着力加强城镇建设。牢固确立规划的龙头地位，城镇一切建设都必须服从规划。加快中心城市、县城、集镇三级城镇体系建设，按照“一江两岸、提升江南、重心北移”的总体布局，着力推进江北新区建设步伐，加快汉江三桥、安康大道、滨江大道建设进度，开工建设花园大道、瀛湖干道和汉江一桥北桥头交通枢纽工程，启动汉滨区级机关北迁和大学园区的设计、进点工作，抓紧市直有关部门北迁的定点及开工建设。加快江南城区综合改造，开工建设桥西广场、香溪大道、西坝防洪综合工程，继续抓好强电入地建设。扎实做好会展中心、汉江四桥等项目的前期准备工作。调整土地供应，抑制房价上涨，加强物业管理，维护业主权益，促进房地产业健康发展。县城建设要突出特色，完善功能，促进生产要素的集聚。集镇建设要因地制宜，完善基础设施，发挥为农服务的作用。巩固背街小巷改造成果，加快居民片区综合改造。扎实开展城镇卫生环境和交通秩序综合整治，下工夫治理群众反映强烈的“脏、乱、差”问题，努力提高城镇管理水平和文明程度。

　　（六）深化改革开放，为突破发展注入活力。全面完成国企改革任务，指导和帮助已改制企业完善经营机制，切实加强对非公有制经济的服务和监管，促进非公有制经济快速增长。积极扶持和推动金融市场建设，不断深化与国家开发银行的金融合作，努力提升政策性银行、国有商业银行、信用社服务当地经济发展的水平。继续完善非经营性国有资产管理体制，切实增强市财信担保、典当公司服务中小企业的能力。加强财税审计工作，依法强化税收征管，壮大地方财力，严格预算约束，严肃财经纪律，加强专款管理，提高使用效益。加强统计对经济运行的监测和分析，认真做好农业普查工作。强化农业生产资料和群众生活必需品价格的监管。积极稳妥的推进乡镇机构改革，深入宣传政策，实施阳光操作，确保公平公正，既要坚定不移地建立起职能明确、运转高效的基层行政管理体制，又要按照政策妥善安置好每一个分流人员。继续完善农村义务教育经费保障机制，巩固和发展农村税费改革成果，积极化解乡村债务，坚决杜绝新的债务。树立开放决定发展的理念，坚持把改善投资环境作为生命线工程来抓，强化社会信用，规范行政行为，提高办事效率，坚决查处影响投资环境的各种行为。建立和完善招商引资新机制，以引进战略投资、大型企业为重点，提高招商引资的针对性，促进资源的优化配置。

　　四、在突破发展中构建和谐安康

　　构建和谐安康，既要从“大社会”着眼，落实到经济、政治、文化、社会建设之中，又要从“小社会”入手，解决人民群众最关心、最直接、最现实的利益问题，实现人与人、人与社会、人与自然的和谐相处。

　　（一）坚持科教兴安战略，优先发展教育事业。认真落实市委、市政府《关于增强科技进步与创新能力促进经济社会突破发展的决定》，紧紧围绕绿色产业的突破发展和社会事业的全面进步，加大科技成果和先进技术的引进、消化和推广力度，建立自主创新的激励机制，激发科技人员的创造热情，加快科技成果转化，提高科技对经济社会的支撑和引领水平。以迎接国家“两基”验收为动力，全面消除全市中小学D级危房，夯实保学控辍责任，提高义务教育普及程度，落实经费保障，加大对农村贫困、边远地区基础教育支持力度，巩固“两基”成果，推动义务教育均衡发展。合理调整教育结构，努力扩大高中规模。大力发展职业教育，以就业为目标，使初高中毕业未升学的学生都能够接受职业技术教育，成为有一技之长的建设者。办好高等教育，积极支持安康学院、职业技术学院建设。推动特殊教育、幼儿教育、民办教育健康发展。

　　（二）加强医疗卫生工作，提高人民健康水平。突出公共医疗卫生公益性质，健全公共卫生防治体系，建立健全县乡村三级医疗服务网络，不断提高重大疾病预防控制和医疗救治能力。切实加强农村卫生工作，继续抓好以防氟砷为重点的地方病防治，新型农村合作医疗由四县试点扩展到全市十县区，扎实抓好乡镇卫生院、村卫生室的建设和设施装备，加大农村卫生人才培养力度。构建社区卫生服务网络，在汉滨区实行城市社区卫生服务保障制度，逐步建立覆盖城乡的基本医疗保健制度。在巩固提高儿童计划免疫工作的基础上，加强母婴保护，提高住院分娩率，对农村纳入低保的贫困母亲住院分娩给予补助。进一步完善单病种最高限价等改革，着力解决群众看病难、看病贵的问题。依法打击非法行医和销售假药，切实加强食品药品市场监管。加强医德医风建设，有效遏制重大医疗事故的发生，严肃追究医疗事故责任。依法维护医院正常的医疗秩序，严厉打击冲击医院等公共服务场所的不法行为。要坚持稳定低生育水平的方针，把精力用在基层基础工作上，切实加强流动人口的管理，巩固“三清三查”成果，严肃“一票否决”、“黄牌警告”制度，夯实计划生育领导、部门和基层责任，切实提高计划生育工作的质量和水平。

　　（三）加快发展文化事业，满足群众文化需求。把社会主义核心价值体系作为建设和谐文化的根本。大力弘扬社会主义荣辱观，广泛开展和谐社区、和谐机关、和谐家庭创建活动。开展丰富多彩的群众文化活动。加大优秀精神文化产品创作力度，深入挖掘紫阳民歌、汉调二簧等非物质文化遗产内涵，加强对汉水文化的研究，打造安康文化品牌。树立新的文化发展观，一手抓文化事业发展，一手抓文化产业培育。加大县、乡镇、村文化设施建设投入，加强文化市场监管，净化校园周边环境，高度重视未成年人的思想道德建设。广播电视等媒体要出精品节目、造品牌栏目，营造和谐向上的舆论氛围。广泛开展全民健身活动，实施“农民健身工程”，扶持修建70个村、30个乡镇和社区体育活动场地，不断满足人民日益增长的精神文化需求。

　　（四）大力推进生态建设，促进人与自然和谐相处。保护安康的青山碧水、蓝天白云，是和谐建设的重要内容。要从生态工程建设入手，以大瀛湖生态治理为示范，继续抓好退耕还林、天然林保护、荒山造林、小流域治理和生态移民工程，造林50万亩，治理水土流失500平方公里，生态移民和扶贫搬迁14500人。加快江南污水处理厂二期工程建设，完成城区雨污分流管网改造，力争白河县城垃圾、污水处理工程立项建设。针对危害人民群众健康的突出问题，加大对污染的专项治理力度。积极展开对生态环境建设和南水北调供水补偿机制的研究。认真落实节能降耗减排各项措施，使经济社会发展步入环境友好型、资源节约型轨道。

　　（五）更加关注群众疾苦，切实维护群众利益。以人为本，关注民生，维护公平，是和谐建设的根本。把扩大就业作为衡量经济增长质量的重要指标，大力发展就业容量大的劳动密集型产业，积极开发公益性岗位，优先安置零就业家庭的失业人员。认真落实就业再就业的税费优惠政策，探索安置复员退伍军人的新途径，引导大学毕业生面向基层就业、依靠市场就业和自主创业。加快社会保障制度建设，进一步扩大养老、失业、工伤、生育等保险制度覆盖面，逐步提高城乡贫困人口最低生活保障补助标准，继续推进农村五保户集中供养，完成70座乡镇敬老院、400个集中供养点建设。认真实施《安康中心城市廉租房建设规划》，确保三年时间解决安康城区2500户困难群众的住房问题,今年解决800户。充分发挥住房公积金在改善中低收入家庭住房条件中的作用。建立健全控辍保学责任制和贫困学生救助制度，依法保障适龄儿童全部入学。继续实施贫困家庭大学生入学资助措施。重视流浪儿童、孤残儿童、留守儿童的基本生活与教育成长，积极争取建设市儿童福利院。对52000绝对贫困人口的吃饭、穿衣、住房、入学、医疗等实施特殊帮扶政策，努力改善他们的生活状况和生存条件。切实维护失地农民的利益，重视解决他们的长远生计。认真执行水库移民后期扶持政策，促进库区和移民安置区经济社会健康发展。积极发展慈善公益和残疾人事业。重视做好红十字会工作。加大对弱势困难群体的法律援助。

　　（六）切实维护社会稳定，高度重视安全生产。清醒认识加速发展期和矛盾凸现期出现的新情况、新问题，统筹协调各种利益关系。建立健全科学有效的利益协调机制、诉求表达机制、矛盾调处机制和权益保障机制，注意运用法律、政策、经济、行政等手段解决各类社会矛盾。加强人民来信来访工作，落实信访工作责任制，加大积案要案的办结力度。完善应急管理机制，提高政府应对自然灾害、事故灾难、公共卫生事件和社会安全事件的能力。深入开展“五五普法”，切实落实社会治安综合治理措施，依法打击各类犯罪活动。以提高公众安全感为目标，加强社会治安防控体系建设，深入开展“平安安康”创建活动，强化公安基层基础工作，努力改善技术装备，为人民群众创造安居乐业的治安环境。进一步重视少数民族聚集区的经济发展，巩固和发展平等、团结、互助、和谐的社会主义民族关系。依法加强宗教事务管理，积极引导宗教与社会主义社会相适应。重视国防后备力量建设，深入开展“双拥”活动。切实加强对台工作。全面落实安全生产责任制，强化交通、矿山、危险化学品等高危行业的监管整治，建立安全生产长效管理机制，坚决遏制重特大事故发生。

　　和谐社会建设是一项系统工程，是一个渐进的过程，不可能一蹴而就。必须分清轻重缓急，防止急于求成，把有限的财力更多的用于困难群体，雪中送炭、体现公平。

　　五、进一步加强政府自身建设

　　实施突破发展，构建和谐安康，对政府自身建设提出了新的更高要求。我们要在科学发展观的指导下，更新行政理念，转变政府职能，狠抓落实工作，努力建设团结和谐、务实高效、勤政廉洁、充满活力、人民满意的政府。

　　（一）坚持科学行政。党的执政能力和人民代表大会决议的执行，主要靠政府的行政能力来体现。科学发展观创新了政府的行政理念和工作方式，要求我们必须从传统的行政模式转向科学行政。以法为度是科学行政的基础。政府及全体组成人员要带头学法、守法、用法，坚决摒弃不懂法，信奉权力的官本位意识；不依法，滥用职权的人治行为；不用法，随心所欲的长官意志。依法管理社会事务，调解社会矛盾，自觉接受法律约束和监督，把行政行为规范在法律许可的范围之内。以人为本是科学行政的目标。政府只能为人民谋利益，决不能谋一己之利，为少数人谋利，为本部门谋利，更不能与民争利。制定决策要立足于长远利益、多数人利益和困难群体利益，用社会公平和人的全面发展作为施政的评判标准。求真务实是科学行政的方法。政府工作必须坚持“三个有利于”标准，坚持实事求是的思想路线，反对脱离群众的官僚主义、主观主义、形式主义，崇尚实干，力戒空谈，讲求行政决策的科学性，行政工作的高效率，行政运行的低成本。

　　（二）加快职能转变。经济调节、市场监管、社会管理、公共服务的四大职能都需要政府改进服务工作来实现，转变政府职能实质上就是建设服务型政府。要切实增强服务能力，改进服务工作，创造发展环境。凡能够取消的行政审批坚决取消，已取消的严禁变相或变通恢复，坚决纠正一些部门和工作人员权力寻租、吃拿卡要。各级政府要舍得“削自己的权”，把管不了管不好的事，下决心交给市场和中介机构；舍得“减自己的利”，坚决纠正公共权力部门化，狠刹巧立名目，以罚代法，向企业伸手；舍得“拆自己的庙”，压缩机构，精简冗员，提高服务效能。充分发挥市场在资源配置中的主导作用，政府工作人员不得直接参与微观经济活动，不得干预企业生产经营，不得以任何形式干扰市场资源的配置。要心系群众、心操基层，放下架子、真诚服务，不打扰民众、不折腾基层、不骚扰企业。

　　（三）狠抓落实工作。抓落实是政府的基本职责，是检验行政能力和工作绩效的标尺，政府工作的一切成效无不来自于落实，群众对我们的意见也主要反映在落实上。责任不清、推诿扯皮、华而不实、消极应付、无所作为、奖罚不明成为落实工作中的顽疾，必须下决心根治。要进一步完善抓落实的机制，从领导机关到基层单位，从领导干部到工作人员，从各级政府到工作部门，都必须把工作任务、质量标准、时限要求落到实处。对重大项目、重点产业、重要工作，实行第一责任人制度，责权到位，定期公示、专项督查，严格奖罚、论功行赏，激发广大干部的发展热情和创造活力。高度重视重点项目建设领军人物和工业经济人才的培养，锤炼一批熟悉经济工作的行家里手。用“三苦”精神改造我们的作风，下茬整治文风、会风，切实改进政风、行风。始终牢记“两个务必”，弘扬艰苦创业，厉行勤俭节约，狠刹奢侈浪费。认真落实廉政建设责任制，积极推进惩防体系建设，严格执行廉洁自律各项规定，继续加大商业贿赂治理力度，强化对权力运行的制约和监督，把廉政建设的各项任务融入政府工作之中。自觉接受人大的工作监督、政协的民主监督、媒体的舆论监督和人民的公众监督，善于吸收人民群众的批评、意见和建议，正视问题，改进工作，减少失误，防止闪失。依法加强公务员队伍的建设和管理，做到言行一致、令行禁止、政令畅通，提高政府的执行力和公信力。

　　今日安康，已经站在一个新的起点上，实施突破发展、构建和谐社会的神圣使命历史的落在我们肩上，295万人民的热切期望，必将化作前进征程上的巨大力量，我们要励精图治、众志成城，坚定有所作为的意志，作出无愧于时代、无愧于人民的回答！

　　各位代表，新的征程任重道远，新的形势催人奋进。让我们紧密团结在以胡锦涛为总书记的党中央周围，在科学发展观的指引下，矢志艰苦奋斗，勇于开拓创新，努力把突破发展的宏伟蓝图变为富民强市的美好现实！
